# Lessons from bacteriophages part 1: Deriving utility from protein structure, function, and evolution

**DOI:** 10.1371/journal.ppat.1006971

**Published:** 2018-05-17

**Authors:** Kunica Asija, Carolyn M. Teschke

**Affiliations:** Department of Molecular and Cell Biology, University of Connecticut, Storrs, Connecticut, United States of America; University of Kentucky, UNITED STATES

## A historical introduction to research using phages

Historically, some of the most fundamental discoveries of modern molecular biology were revealed by examination of phage-infected cells [1]. Some examples include the use of bacteriophage T2 to show that DNA, not protein, was the genetic material by Hershey and Chase in 1952 [2]. Crick and colleagues showed that codons are degenerate and encode for single amino acids by making use of T4 bacteriophage. Brenner and colleagues discovered that mRNA acts as the messenger in relaying the information from DNA to ribosomes with the aid of bacteriophage T2 [3]. Szybalski and colleagues demonstrated the semiconservative mechanism of DNA replication using λ phage [4]. Restriction modification, a bacterial innate immune system that evolved as protection against invading mobile genetic elements such as plasmids and phages, was discovered in bacteriophage-infected cells. Restriction mapping was crucial for the completion of the human genome project. The mechanism of action of heat shock genes—which encode for molecular chaperones such as *dnaK*, *dnaJ*, and *grpE*, as well as the *groE* gene locus, which encodes for the molecular chaperones GroES and GroEL—were discovered in λ phage–infected *Escherichia coli* [5]. The first capsid assembly inhibitor, the fluorescent dye 4,4’–bis(1-anilinonaphthalene-8-sulfonic acid) (bisANS), was discovered in experiments investigating assembly of phage P22 [6]. These are just some of the examples of contributions made by bacteriophage research in the past few decades. Here, we discuss additional discoveries derived from studies of phages.

## Insights from phage capsid protein structures and assembly

Dennis Bamford and colleagues made a key observation that links eukaryotic, archaeal, and prokaryotic viruses: Despite the lack of sequence homology, viruses from all domains of life have capsid proteins that fall into four structural lineages [7]. These lineages are likely due to divergent evolution from common viral progenitors [7]. For example, the picornavirus-like lineage has a characteristic jelly roll or β-barrel roll fold in its capsid protein. This fold is seen in plus-sense single-stranded RNA (ssRNA), ssDNA, double-stranded RNA (dsRNA), and dsDNA viruses and phages, although it is most common in RNA viruses [7]. As another example, the dsDNA phages and herpesviruses have a conserved coat protein fold known as the Hong Kong 97 (HK97)-fold. We call this “nature’s favorite building block” due its common occurrence in the dsDNA class of viruses, which are the most abundant entities on Earth [8]. With its perfect wedge shape that can readily form the hexons and pentons requisite for building an icosahedral capsid, the HK97-fold can be used to build an amazing array of capsids with triangulation numbers ranging from 1 to 52, yielding capsid diameters of 25 nm to 160 nm [9]. While the viruses in the HK97-fold lineage have coat proteins that share less than 10%–15% sequence similarity, they have similar assembly mechanisms. For instance, several parallels can be drawn in the assembly processes of Herpesviridae and members of the Caudoviridae family comprising the tailed bacteriophages, including P22, T4, Φ29, and HK97. These include the formation of an unstable procapsid intermediate, the use of an internal scaffolding protein (or delta domain) to aid in the formation of the procapsid that is absent from the mature virion, and the use of a unique portal vertex for the uptake of DNA into the empty procapsid during maturation [10]. In case of T4 and HK97 phages, the scaffolding protein is proteolytically cleaved during the maturation process, a feature seen in herpesvirus as well [11]. These proteases, belonging to the procapsid protease superfamily, are evolutionarily related [8, 12].

**Fig 1:**
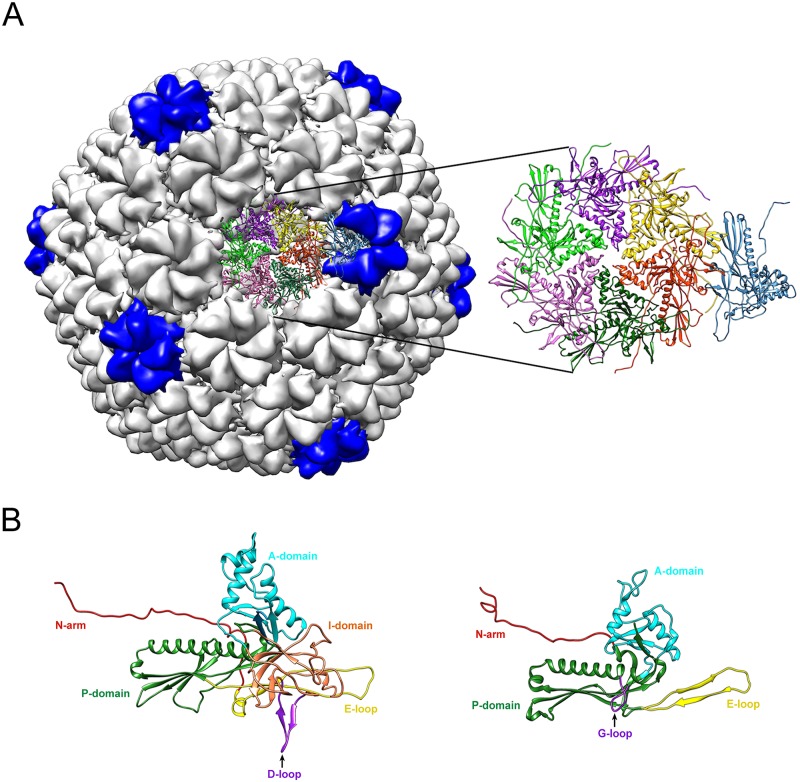
(A) Capsid structure of bacteriophage P22. The left side of panel A shows the mature P22 capsid. The pentons are highlighted in blue, and hexons are shown in gray. The asymmetric unit (seven subunits) of the capsid is represented in ribbon format in the center of the capsid. The asymmetric unit is magnified and shown on the right of the panel. (B) Ribbon diagrams of coat monomers of P22 (left) and HK97 (right). The A-domains, P-domains, E-loops, and N-arms have been highlighted in cyan, green, yellow, and red, respectively. The I-domain of P22 coat protein has been highlighted in coral. The arrows indicate the D-loop (purple) of the monomer of P22 (left) and the G-loop (purple) on the monomer of HK97 (right).

The structural homology observed in coat proteins can be generalized and applied to other structurally related viruses. If we begin by considering the phage HK97 coat protein (Fig 1), the G-loops interact with the E-loop of an adjacent subunit to form a salt bridge that stabilizes the capsomer interactions in the procapsid across a 2-fold axis of symmetry [13]. In phage P22, an extra domain (I-domain) is inserted within the A-domain of the HK97 core of the coat protein (Fig 1) [14, 15]. The D-loops of the I-domain make stabilizing contacts across the 2-fold axis of symmetry with the adjacent subunit, similar to the G-loops (within the P domain) in the HK97 coat protein [16]. The roles of these loops in the proper procapsid formation have been elucidated experimentally in both HK97 and P22: Aberrant structures such as tubes result from substitutions in these loops. A similar theme can be seen with the E-loop of the HK97 fold, again with slight variations as we compare coat proteins. In the HK97 coat protein, E-loop interactions stabilize the capsids through making covalent isopeptide crosslinks with the P-domain of the adjacent subunit [13], as well as ionic interactions with G-loops [17]. In phage T4’s coat protein, a domain is inserted into the E-loop. This inserted domain interacts with an adjacent subunit, stabilizing the capsid in a fashion analogous to the crosslinks in HK97 [18]. Structural evaluation of the phage P22 capsid indicates that the E-loop makes salt bridges with the spine helix of the neighboring subunit to stabilize the capsid [15]. Overlay of cryoEM density maps of herpes simplex virus-1 (HSV-1) base domain with HK97 coat protein highlights the importance of the E-loop in HSV-1 capsid as well. The E-loop of HSV-1 contacts the adjacent subunits at multiple points, including the spine helix and the middle and upper domains. The density maps also show that the E-loop of the HSV-1 capsid interacts with the N-arm of an adjacent subunit, similar to that observed in phage HK97 [19]. These data indicate that the E-loop plays a crucial role in making stabilizing contacts with other subunits, and it appears to be conserved between structurally similar phages and viruses.

## Phages and vaccines

The repeated use of the same subunit to build an icosahedral capsid, and the high stability of phage capsids, has made phages ideal vehicles for vaccine development. Phage particles with displayed antigens and virus-like particles (VLPs) robustly provoke immune responses. When phage F1 was conjugated with B2.1 peptide, the immune response generated in mice was much stronger than when the peptide was conjugated with ovalbumin [20], and when the filamentous bacteriophage *fd* was conjugated with peptide epitopes, both humoral and cellular immune responses were elicited by activating major histocompatibility complexes (MHC) I and II after being taken up by lymphocytes [21]. In an additional example, λ phage decorator protein gpD (the product of gene D) fused with fragments of aspartate β-hydroxylase, a protein overexpressed in hepatocarcinoma cells, provoked a strong antitumor immune response in mice [22].

**Fig 2:**
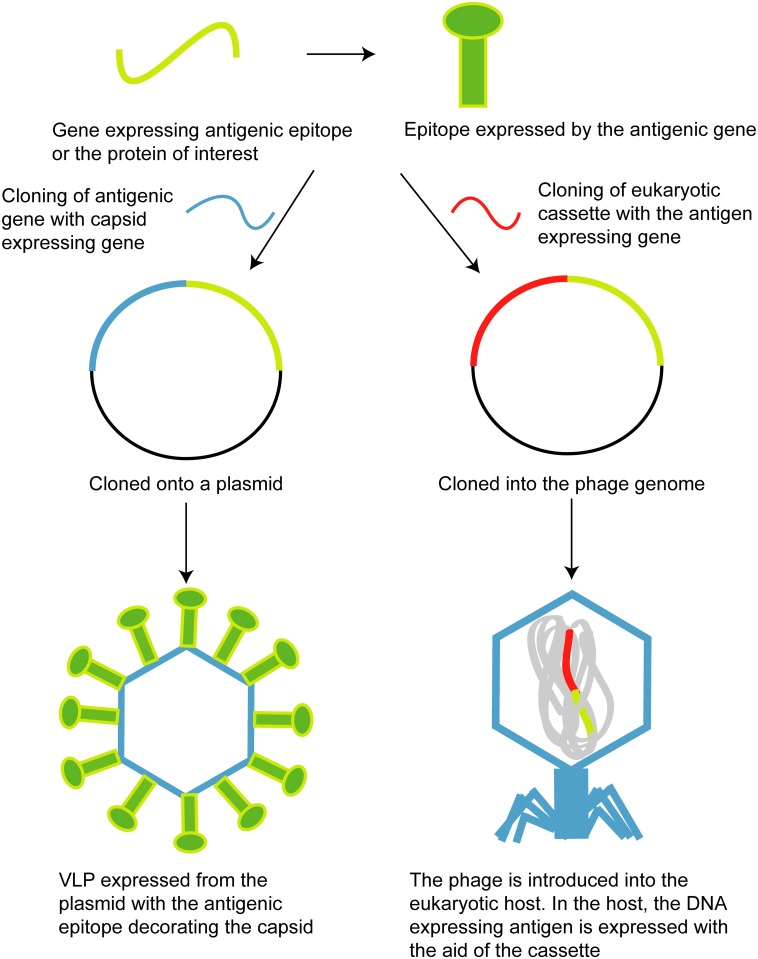
Phage use in vaccine design and delivery. The left panel shows the use of a VLP to express an antigenic gene product on its capsid. The right panel shows an antigenic gene being cloned into the phage genome and its subsequent use as a DNA vaccine carrier. VLP, virus-like particle.

VLPs are also used for vaccines. VLPs are composed of only the protein structural units of a virus and lack genetic material, thereby rendering them noninfectious (Fig 2). These particles illicit a strong immune response of both the innate as well as the adaptive immune systems [23]. The size of VLPs ranges from 20–200 nm, allowing them to be endocytosed by different cells in the human body, including antigen-presenting cells such as dendritic cells and macrophages [23]. Both RNA and DNA phages (MS2, PP7, Qβ, P22) are being used to generate VLPs for vaccines [24]. VLPs from the RNA bacteriophage Qβ are being tested as vaccine carriers against Alzheimer disease, malignant melanomas, and type II diabetes mellitus, all of which are in different phases of clinical trials [25].

Phages are being engineered for DNA vaccines, in which the gene encoding the antigenic epitope is cloned into the phage genome but is under the control of a eukaryotic expression cassette (Fig 2). A eukaryotic expression cassette is composed of a promoter, an open reading frame (ORF), and a polyadenylated 3’ untranslated region (UTR). Phage DNA vaccines are easily taken up by antigen-presenting cells, and the phage capsids protect the DNA from nucleases. About 15–20 kb expression constructs can be carried within phages such as λ, which means multiple vaccines can be cloned in the phage genome at once. A DNA vaccine for the small surface antigenic (HBsAg) of hepatitis B virus has been developed using λ phage. This phage vaccine improves upon the previously used hepatitis B vaccines, which failed to elicit a strong immunogenic response. The phage vaccine has been successfully tested on rabbits, in which it is highly immunogenic, and is now awaiting human trials [26].

## Insights based on phage tail machinery

Evolutionary links also can be seen in the tail machinery of bacteriophages and the type VI secretion system (T6SS) of bacteria [27]. The T6SS system is used by many Gram-negative α-, β-, and γ-proteobacteria to deliver proteins to other bacteria and eukaryotes and has features that are structurally analogous to contractile myophage tails. The T6SS system is comprised of three structural components: the inner tube, sheath, and baseplate. The hemolysin coregulated protein (HCP), a crucial component of the T6SS, spontaneously assembles into hexamers to form the tube. The tip of the tube contains a trimer of the VgrG protein, another hallmark protein of the T6SS. A sheath wraps around the tube to form an inverted phage tail–like structure [28] and provides the tube with a contractile function to release effector molecules. HCP protein is structurally related to the tail tube protein (gpV) of λ bacteriophage [29]. The VgrG protein shares sequence as well as structural similarity to the tail needle machinery of the T4 bacteriophage. It also shares functional homology, since it is responsible for puncturing target cells [27, 30]. The general mechanism of assembly for the two systems is similar in the need for a baseplate and the use of the tube as a template for the polymerization of the sheath [31].

The R- and F-type pyocins produced by *Pseudomonas aeruginosa* are structurally similar to phage tails and are thought to have evolved from defective phages owing to the high sequence homology and the presence of a lysis cassette in the pyocin gene cluster [32, 33]. The R-type pyocins share a high similarity to the contractile, nonflexible tail structures of the P2 phages, whereas the F-type pyocins are related to the flexible and noncontractile λ phage tail [32]. As a final example of phage proteins being absconded for alternative uses by other organisms, Photorhabdus virulence cassettes (PVCs) have been found in the insect pathogen *Photorhabdus* and in the amber disease-associated plasmid (pADAP) of *Serratia entomophila*. PVCs contain phage-derived toxin genes that inhibit the larvae of beetles from feeding [27]. Apart from gene products that confer insect larvae with antifeeding activity, PVCs also encode a bacteriocin that is structurally similar to the R-type pyocin [27]. This shows how widespread the phage genes are and that these genes are likely just a fraction of the ones that get fixed in the host chromosome or their extra chromosome elements.

## Conclusion

Time and again, bacteriophages have helped further scientific progress in biology. Evolutionary links can be seen between viruses such as phage P22 and HSV-1 and also between bacterial viruses and their hosts. This makes bacteriophages ideal as model systems to understand mechanisms such as viral assembly and evolution of pathogenic bacteria. Certainly, further studies will find even more links between viruses infecting hosts from different domains and reveal new relationships between viruses and their host cells.
